# Suppression of colitis-related mouse colon carcinogenesis by a COX-2 inhibitor and PPAR ligands

**DOI:** 10.1186/1471-2407-5-46

**Published:** 2005-05-16

**Authors:** Hiroyuki Kohno, Rikako Suzuki, Shigeyuki Sugie, Takuji Tanaka

**Affiliations:** 1 Department of Oncologic Pathology, Kanazawa Medical University, 1-1 Daigaku, Uchinada, Ishikawa 920-0293, Japan

## Abstract

**Background:**

It is generally assumed that inflammatory bowel disease (IBD)-related carcinogenesis occurs as a result of chronic inflammation. We previously developed a novel colitis-related mouse colon carcinogenesis model initiated with azoxymethane (AOM) and followed by dextran sodium sulfate (DSS). In the present study we investigated whether a cyclooxygenase (COX)-2 inhibitor nimesulide and ligands for peroxisome proliferator-activated receptors (PPARs), troglitazone (a PPARγ ligand) and bezafibrate (a PPARα ligand) inhibit colitis-related colon carcinogenesis using our model to evaluate the efficacy of these drugs in prevention of IBD-related colon carcinogenesis.

**Methods:**

Female CD-1 (ICR) mice were given a single intraperitoneal administration of AOM (10 mg/kg body weight) and followed by one-week oral exposure of 2% (w/v) DSS in drinking water, and then maintained on the basal diets mixed with or without nimesulide (0.04%, w/w), troglitazone (0.05%, w/w), and bezafibrate (0.05%, w/w) for 14 weeks. The inhibitory effects of dietary administration of these compounds were determined by histopathological and immunohistochemical analyses.

**Results:**

Feeding with nimesulide and troglitazone significantly inhibited both the incidence and multiplicity of colonic adenocarcinoma induced by AOM/DSS in mice. Bezafibrate feeding significantly reduced the incidence of colonic adenocarcinoma, but did not significantly lower the multiplicity. Feeding with nimesulide and troglitazone decreased the proliferating cell nuclear antigen (PCNA)-labeling index and expression of β-catenin, COX-2, inducible nitric oxide synthase (iNOS) and nitrotyrosine. The treatments increased the apoptosis index in the colonic adenocarcinoma. Feeding with bezafibrate also affected these parameters except for β-catenin expression in the colonic malignancy.

**Conclusion:**

Dietary administration of nimesulide, troglitazone and bezafibrate effectively suppressed the development of colonic epithelial malignancy induced by AOM/DSS in female ICR mice. The results suggest that COX-2 inhibitor and PPAR ligands could serve as an effective agent against colitis-related colon cancer development.

## Background

Colorectal cancer (CRC) is one of the leading causes of death in the world. This malignancy is also one of the most serious complications of inflammatory bowel disease (IBD), including ulcerative colitis (UC) and Crohn's disease [1]. Long-term UC patients have an increased risk of developing CRC compared with the general population [2]. The precise mechanisms of the IBD-related carcinogenesis process are largely unclear, although it is generally assumed that IBD-related carcinogenesis occurs as a result of chronic inflammation [3].

Several agents, such as folic acid, short chain fatty acid (butyrate), ursodeoxycholic acid, and 5-aminosalicylic acid, have been suggested to be useful for prevention of CRC in UC [4]. Epidemiological studies have shown that prolonged use of aspirin is associated with a reduced risk of CRC [5]. Consistent with these data, several non-steroidal anti-inflammatory drugs (NSAIDs), including cyclooxygenase (COX)-2 inhibitors, suppressed the development of chemically-induced colon carcinomas in rats [6] and intestinal polyps in *Min *mice with a nonsense mutation of the *Apc *gene [7]. In addition, clinical trials have demonstrated that a NSAID sulindac causes regression of adenomas in patients with familial adenomatous polyposis [8]. Nimesulide (4-nitro-2-phenoxymethanesulfonanilide), a selective inhibitor of COX-2, belonging to the sulfonamide class [9], is less ulcerogenic than other NSAIDs [10], and suppresses the formation of aberrant crypt foci (ACF), being putative precancerous lesions of the colon cancer, induced by a colon carcinogen, azoxymethane (AOM) in rats [11]. Moreover, this COX-2 inhibitor could effectively reduce the development of intestinal polyps in *Min *mice [12].

Peroxisome proliferator-activated receptors (PPARs?) are ligand-activated transcription factors, belonging to the nuclear hormone receptor superfamily [13]. Three PPAR isotypes, PPARα, PPARδ(β), and PPARγ, have been identified. PPARγ is highly expressed in fat tissue, and play important roles in adipocyte differentiation and lipid storage [14]. PPARγ is also expressed in a number of epithelial neoplasms, such as cancers in colon, breast, and prostate [13]. PPARγ ligands, including thiazolidinediones (troglitazone and rosiglitazone) and tyrosine analogue (GW7845), can induce apoptosis and adipogenic differentiation, and inhibit tumor growth both *in vitro *and *in vivo *studies [15-17]. We previously reported that pioglitazone, bezafibrate or troglitazone in diet are able to suppress ACF formation induced by dextran sodium sulfate (DSS)/AOM in the rat colon [18]. Osawa *et al*. [19] confirmed our findings by demonstrating that ligands for PPARγ (troglitazone, rosiglitazone, and pioglitazone) suppress the occurrence of colonic tumors in mice initiated with AOM. Niho *et al*. [20] also demonstrated that ligands for PPARα (bezafibrate) and PPARγ (pioglitazone) suppress intestinal polyp formation in *Apc*-deficient mice. Moreover, PPARγ could suppress β-catenin levels and colon carcinogenesis during the early steps of tumor formation [21]. On the other hand, high doses of troglitazone and rosiglitazone can promote polyp formation in the *Min *mouse colon [22,23].

For understanding the pathogenesis of IBD and IBD-related CRC, several animal models have been established. Most used is a mouse model with DSS [24]. Modifying effects of several xenobiotics on IBD-related colon carcinogenesis were reported [25] in animal models of IBD. However, the colitis model using DSS with or without carcinogen needs to a long period and repeated administration of DSS to induce colitis and colitis-related CRC. Recently, an endogenous anti-inflammatory PPARγ pathway was suggested in the intestine, which was found in PPARγ-deficient mice [26,27]. To search novel and effective chemopreventive agents against IBD-related CRC, we recently have developed a novel colitis-related CRC mouse model, in which large bowel adenocarcinomas occur within 20 weeks and their histology and biological characteristics are resemble to those found in human cased [28]. As a part of our search for a safer chemopreventive agent for colitis-related colon cancer, in the present study we examined the chemopreventive ability of nimesulide, troglitazone, and bezafibrate using our mouse colon carcinogenesis model for colitis-related colon carcinogenesis [28].

## Methods

### Animals, chemicals and diets

Female Crj: CD-1 (ICR) (Charles River Japan Inc., Tokyo, Japan) aged 5 weeks were used in this study. They were maintained at Kanazawa Medical University Animal Facility according to the Institutional Animal Care Guidelines. The mice were quarantined for the first 7 days then randomized by body weight into experimental and control groups. All animals were housed in plastic cages (five or six mice/cage) with free access to drinking water and a pelleted basal diet, CRF-1 (Oriental Yeast Co., Ltd., Tokyo, Japan), under controlled conditions of humidity (50 ± 10%), light (12/12 hour light/dark cycle) and temperature (23 ± 2°C). A colonic carcinogen AOM was purchased from Sigma Chemical Co. (St. Louis, MO, USA). DSS with a molecular weight of 40,000 was purchased from ICN Biochemicals, Inc. (Aurora, OH, USA). DSS for induction of colitis was dissolved in distilled water at a concentration of 2% (w/v). Nimesulide was purchased from Sigma Chemical Co. (St. Louis, MO, USA). Troglitazone and bezafibrate were kindly supplied by Sankyo Co. (Tokyo, Japan) and Kissei Pharmaceutical Co. (Matsumoto, Japan), respectively. Experimental diet containing nimesulide (0.04%, w/w), troglitazone (0.05%, w/w) or bezafibrate (0.05%, w/w) was prepared every week by mixing the respective compound in powdered basal diet CRF-1. The dose levels were determined on the basis of previous studies [18,29].

### Experimental procedure

**Figure 1:**
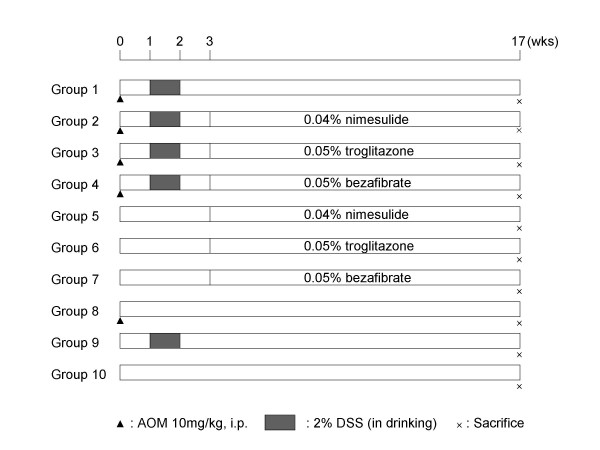
Experimental protocol. Arrows, AOM 10 mg/kg body weight, i.p. injection; densely cross-hatched bars, 2% dextran sodium sulfate (DSS) in drinking water; open bars, basal diet and tap water; crosses, death.

A total of 71 female ICR mice were divided into 7 experimental and control groups (Figure 1). Mice in groups 1 through 4 were given a single intraperitoneal injection of AOM (10 mg/kg body weight). Starting one week after the AOM injection, animals in group 1 were administered to 2%DSS in drinking water for 7 days, and then followed without any further treatment for 15 weeks. Mice in groups 2, 3, and 4 were fed the diets containing 0.04% nimesulide, 0.05% troglitazone, and 0.05% bezafibrate, respectively, for 14 weeks, starting 1 week after the stop of DSS administration. Animals in groups 5, 6, and 7 were respectively given the diets containing 0.04% nimesulide, 0.05% troglitazone, and 0.05% bezafibrate alone for 14 weeks. Group 8 was given a single dose of AOM. Group 9 was given 2% DSS for 7 days. Group 10 consisted of untreated mice. All animals were sacrificed at the end of the study (Week 17) by ether overdose. Their large bowels were flushed with saline, excised, measured their length (from ileocecal junction to the anal verge), cut open longitudinally along the main axis, and then washed with saline. The large bowels were macroscopically inspected, cut, and fixed in 10% buffered formalin for at least 24 hours. Histopathological examination was performed on paraffin-embedded sections after hematoxylin and eosin (H & E) staining. Colonic mucosal dysplasia (mild- and severe-graded) was diagnosed according to the criteria described by Ridell *et al*. [30] and Pascal [31]. Colonic neoplasms were diagnosed according to the description by Ward [32].

### Immunohistochemistry

Immunohistochemistry for the proliferating cell nuclear antigen (PCNA), apoptotic nuclei, β-catenin, COX-2, inducible nitric oxide synthase (iNOS), and nitrotyrosine was performed on 4-μm-thick paraffin-embedded sections from colons of mice in each group by the labeled streptavidin biotin method using a LSAB KIT (DAKO Japan, Kyoto, Japan) with microwave accentuation. The paraffin-embedded sections were heated for 30 min at 65°C, deparaffinized in xylene, and rehydrated through graded ethanol at room temperature. A 0.05 M Tris HCl buffer (pH 7.6) was used to prepare solutions and for washes between various steps. Incubations were performed in a humidified chamber. For the determination of PCNA-incorporated nuclei, the PCNA-immunohistochemistry was performed according to the method described by Watanabe *et al*. [33]. Apoptotic index was also evaluated by immunohistochemistry for single stranded DNA (ssDNA) [33]. Sections were treated for 40 min at room temperature with 2% BSA and incubated overnight at 4°C with primary antibodies. Primary antibodies included anti-PCNA mouse monoclonal antibody (diluted 1:50; PC10, DAKO Japan), anti-ssDNA rabbit polyclonal antibody (diluted 1:300, DAKO Japan), anti-β-catenin mouse monoclonal antibody (diluted 1:1000, Transduction Laboratories, Lexington, KY, USA), anti-COX-2 rabbit polyclonal antibody (diluted 1:50, IBL Co., Ltd., Gunma, Japan), anti-iNOS rabbit polyclonal antibody (diluted 1:1000, Wako Pure Chemical Industries, Ltd., Osaka, Japan), and anti-nitrotyrosine rabbit polyclonal antibody (diluted 1:500, Upstate Biotechnology, Lake Placid, NY, USA). To reduce the non-specific staining of mouse tissue by the mouse antibodies, a Mouse On Mouse IgG blocking reagent (Vector Laboratories, Inc., Burlingame, CA, USA) was applied for 1 h. Horseradish peroxidase activity was visualized by treatment with H_2_O_2 _and 3,3'-diaminobenzidine for 5 min. At the last step, the sections were weakly counterstained with Mayer's hematoxylin (Merck Ltd., Tokyo, Japan). For each case, negative controls were performed on serial sections. On the control sections, incubation with the primary antibodies was omitted.

Intensity and localization of immunoreactivities against all primary antibodies used were examined on all sections using a microscope (Olympus BX41, Olympus Optical Co., Ltd., Tokyo, Japan). The PCNA and apoptotic indices were determined by counting the number of positive cells among at least 200 cells in the lesion, and were indicated as percentages. Each slide for β-catenin, COX-2, iNOS, and nitrotyrosine immunohistochemistry was evaluated for intensity of immunoreactivity on a 0 to 4+ scale. The overall intensity of the staining reaction was scored with 0 indicating no immunoreactivity and no positive cells, 1+ weak immunoreactivity and < 10% of positive cells, 2+ mild immunoreactivity and 10–30% of positive cells, 3+ moderate immunoreactivity and 31–60% of positive cells, and 4+ strong immunoreactivity and 61–100% of positive cells.

### Statistical analysis

All measurements were compared by Student's *t*-test, Welch's *t*-test or Fisher's exact probability test for paired samples.

## Results

### General observation

Bloody stool was noted in a few mice received 2% DSS and their body weight gains were slightly decreased during the period of the treatment. However, thereafter no such clinical symptoms were observed. After Week 12, anal prolapsus due to the tumor development in the distal colon was found in a few mice treated with AOM and 2% DSS (group 1). The body weights and lengths of large bowel of mice in all groups at the end of the study are shown in Table 1. The mean body weights of groups 2 (AOM/DSS/0.04% nimesulide, *P *< 0.005), 3 (AOM/DSS/0.04% troglitazone, *P *< 0.02), and 4 (AOM/DSS/0.05% bezafibrate, *P *< 0.05), were significantly higher than that of group 1 (AOM/DSS). The mean body weight of group 9 (DSS alone, *P *< 0.05) was significantly lower than that of group 10 (untreated). The mean liver weights of mice in groups 3 (*P *< 0.05) and 4 (*P *< 0.01) were significantly greater than that of group 1. However, there were no pathological alterations suggesting toxicity of test compounds in the liver, kidneys, lung, and heart of mice (data not shown).

### Pathological findings

**Figure 2:**
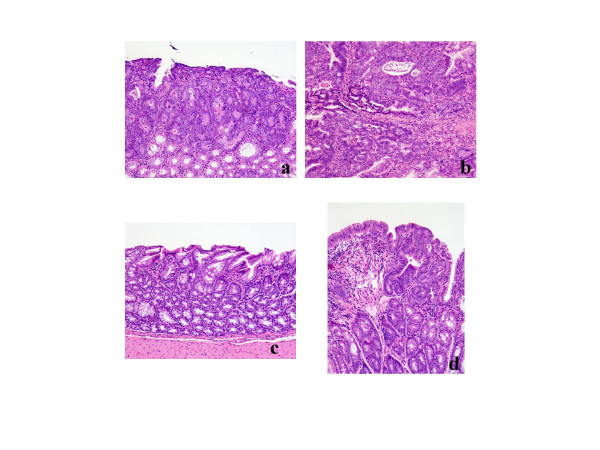
Histopathology of colonic lesions in mice of group 1. (a) adenoma, (b) adenocarcinoma, (c) mild dysplasia, and (d) severe dysplasia. Hematoxylin and eosin stain. Original magnifications, × 10.

Macroscopically, nodular, polypoid or caterpillar-like tumors were observed in the middle and distal colon of mice in groups 1 through 4. They were histologically tubular adenoma (Figure 2a) or well-/moderately-differentiated tubular adenocarcinoma (Figure 2b). Dysplasia (Figure 2c–e) was also developed in mice of these groups. Animals of groups 5–10 did not have large bowel neoplasms and dysplasia. The incidences and multiplicity of colon neoplasma are shown in Table 2. Group 1 (AOM/DSS) induced 100% incidence of colon adenocarcinomas with a multiplicity of 3.0 ± 1.8. The incidences of colorectal adenocarcinomas in groups 2 (AOM/DSS/0.04% nimesulide), 3 (AOM/DSS/0.05% troglitazone), and 4 (AOM/DSS/0.05% bezafibrate) were significantly smaller than that of group 1 (*P *< 0.01, *P *< 0.01 and *P *< 0.05, respectively). The multiplicities of colon adenocarcinomas in groups 2 and 3 were also significantly lower than that of group 1 (*P *< 0.005 and *P *< 0.05, respectively). While the multiplicity of colon adenocarcinoma of group 4 (AOM/DSS/0.05% bezafibrate) was smaller than group 1, the difference was insignificant. In this study, mucosal ulcer with or without focal dysplasia (Figure 2c–e) were also found in the distal colon of mice in groups 1 through 4. The incidences and multiplicity of colonic ulceration and dysplasia are shown in Table 3. The incidences and multiplicities of colorectal mucosal ulcer and dysplasia of groups 2, 3, and 4 were smaller than group 1, but the differences did not reach to statistical significance.

### Immunohistochemistry for PCNA, ssDNA, β-catenin, COX-2, iNOS and nitrotyrosine in colonic adenocarcinoma

**Figure 3:**
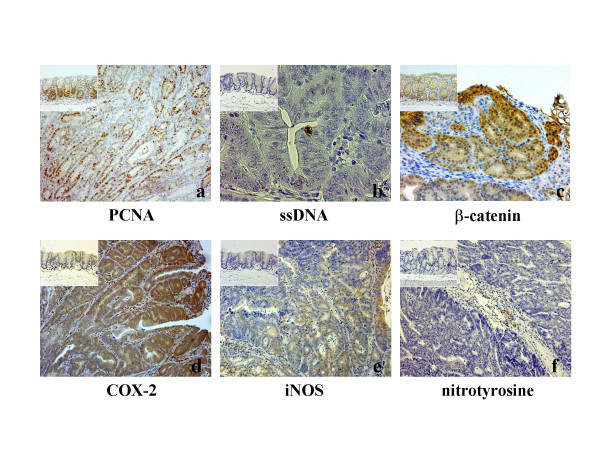
Immunohistochemistry of PCNA, ssDNA, β-catenin COX-2, iNOS, and nitrotyrosine in mice of group 1. (a) PCNA immunohistochemistry, (b) ssDNA immunohistochemistry, (c) β-catenin immunohistochemistry, (d) COX-2 immunohistochemistry, (e) iNOS immunohistochemistry, and (f) nitrotyrosine immunohistochemistry. Original magnifications: a, d, e, f, × 10; c, × 20; b, × 40. Insets, immunohistochemistry staining for each antibody in the mouse colon from group 10 (Original magnifications, × 10).

As summarized in Table 4, PCNA-labeling index (Figure 3a) of colonic adenocarcinomas developed in groups 2, 3, and 4 was significantly smaller than group 1 (*P *< 0.001). Apoptotic index measured by ssDNA immunohistochemistry (Figure 3b) in groups 2, 3, and 4 was significantly greater than group 1 (*P *< 0.001).

Strong β-catenin expression was seen in the nucleus and cytoplasm of adenocarcinoma cells (Figure 3c). Although the intensity was relatively weaker than carcinoma cells, adenoma cells showed positivity for β-catenin in their cytoplasm and cell membrane. β-catenin immunoreactivity was also found in the cell membrane and cytoplasm of dysplastic cells. Non-lesional cryptal cells showed weak positivity of β-catenin in their cell membrane. In the positive cases of COX-2 (Figure 3d), and iNOS (Figure 3e) expression in the dysplasia and adenocarcinoma, the staining pattern was granular and localized to cytoplasm and/or nuclei. Slight immunoreactivity for COX-2 and iNOS was observed in the superficial layers of the non-lesional colonic mucosa and in parts of basal layer in all groups. The expression pattern between COX-2 and iNOS of colorectal adenocarcinomas was well correlated. Furthermore, a positive staining for nitrotyrosine, a marker of nitrosative injury, was mainly observed in mononuclear cells infiltrated in the colonic mucosa (Figure 3f). Neoplastic cells also showed negative or very weakly positive immnoreactivity of nitrotyrosine. Scores for β-catenin, COX-2 and iNOS expression in colonic adenocarcinomas are given in Table 4. β-Catenin expression scores of colorectal adenocarcinomas in groups 2 and 3 were significantly decreased when compared with that in group 1 (*P *< 0.05 and *P *< 0.05, respectively). Scores for COX-2 and iNOS expression of colorectal adenocarcinomas in groups 2, 3, and 4, were significantly smaller than those in group 1 (*P *< 0.001). The scores of nitrotyrosine positivity in groups 2, 3, and 4 were significantly lower than group 1 (*P *< 0.001).

## Discussion

The results of the present work clearly indicated that a COX-2 inhibitor nimesulide and a PPARγ ligand troglitazone effectively inhibited AOM/DSS-induced colitis-related colonic carcinogenesis in mice. Inhibitory effect of nimesulide was superior to that of troglitazone. Bezafibrate also reduced the occurrence of colonic adenocarcinoma, but the ability was relatively weaker than that of nimesulide and troglitazone. The suppressive effects of nimesulide, troglitazone and bezafibrate on the development of colonic adenocarcinoma was well correlated with the inhibition of cell proliferation activity, induction of apoptosis, and lowered immunoreactivity of β-catenin, COX-2, iNOS, and nitrotyrosine in the colonic malignancies. However, no differences on the frequency of dysplastic lesions could be observed among the groups. These data may suggest that the pharmacological classes tested under the present investigation slow down the time course of tumor development rather than completely preventing it.

The pathogenesis of IBD-associated colorectal carcinogenesis is widely believed to involve a step-wise progression from inflamed and hyperplastic epithelia through flat dysplasia to finally adenocarcinoma [30]. IBD-associated colorectal carcinogenesis is probably promoted by chronic inflammation, but the mechanism is still unclear. However, mucosal inflammation may result in colonic carcinogenesis through several proposed mechanisms such as induction of genetic mutations, increased-cryptal cell proliferation, changes in crypt cell metabolism, changes in bile acid enterohepatic circulation, and alterations in bacteria flora [4,34]. These events are considered to promote IBD-associated CRC development. Given the correlation between increased COX-2 expression and colonic carcinoma and/or inflammation, the chemopreventive effects of NSAIDs seem to be mediated, at least in part, by COX inhibition [35]. We [36] and others [29,37] demonstrated that NSAIDs including nimesulide inhibited both colon tumorigenesis and colitis. In the current study, powerful chemopreventive ability of nimesulide was observed in our colitis-related mouse colon carcinogenesis model, suggesting that nimesulide can be applied as an effective chemopreventor of both sporadic and IBD-associated cololectral carcinogenesis.

We previously demonstrated that dietary administration of PPARα and PPARγ ligands inhibits AOM and/or DSS-induced ACF in rodents [38]. In the present study, cancer chemopreventive ability of the PPARγ ligand, troglitazone, or the PPARα ligand, bezafibrate, was found in AOM/DSS-induced mouse colon carcinogenesis model, although their ability was lower than nimesulide. Inhibition of colonic inflammation and decrease in cell proliferation activity by these PPARs ligands might be responsible for their chemopreventive effects on colitis-associated colon carcinogenesis [38]. DNA damage caused by reactive oxygen and nitrogen species may contribute to colitis-related colon tumorigenesis [39]. Several NSAIDs can bind to PPARα and PPARγ [40]. Their anti-inflammatory activities might be mediated through inhibition of COX-1 and/or COX-2. PPARα could suppress COX-2 induction [41]. In addition, immunomodulation by the PPARs ligands might contribute to inhibition of colitis and colon carcinogenesis [42].

Expression and activity of iNOS is increased in colonic mucosa in patients with IBD [43] and colonic adenomas [44]. Several studies using experimental colon carcinogenesis models indicate that chemically induced colon tumors have higher expression and/or activity of iNOS compared with those in their adjacent non-tumorous tissues [12,25]. Numerous iNOS-positive and nitrotyrosine-positive inflammatory cells are observed in non-cancerous colonic mucosa of mice treated with DSS [25]. PPARα [45] and PPARγ [46] involve in inflammation control, and can inhibit iNOS expression [47]. In addition, Rao *et al*. [48] showed that an iNOS-selective inhibitor suppresses AOM-induced colonic ACF development and iNOS activity. Furthermore, nitrotyrosine may originate from the reaction of iNOS generated NO with reactive oxygen species [49] or the myeloperoxidase-dependent pathway [50]. In the present study, we found a positive immunoreactivity for iNOS and nitrotyrosine in the inflamed colon, suggesting the formation of peroxynitrite and other NO-derived oxidants. These results may suggest that one of the mechanisms by which tested agents exert chemopreventive ability might be related to suppression of iNOS activity and/or expression.

Cell proliferation plays an important role in multi-step carcinogenesis [51]. In the colon, the number of cryptal cells is strictly regulated by a balance between cell proliferation and cell death that maintains homeostasis [52]. Changes in cell proliferation and apoptosis are regarded as a common denominator in the pathogenesis of tumor formation [53]. Reduced tumor incidence is generally associated with decreases in cellular proliferation and/or increases in apoptosis [54]. An increased COX-2 expression in CRC [55,56] may confer a survival advantage on cells by inhibition of apoptosis and a change in cellular adhesion to the extracellular matrix [57]. Cancer cells treated with PPARγ ligands induce cell differentiation and apoptosis [14,17]. Recently, Tardieu *et al*. [58] demonstrated that nimesulide increases apoptosis in colonic mucosa of DSS-treated rats. Our findings that nimesulide and troglitazone inhibited cell proliferation activity and induced apoptosis in colorectal mucosa are in accordance with these findings. Thus, cellular responses like cell growth and/or apoptosis to nimesulide and troglitazone may contribute to chemopreventive effects against colon carcinogenesis processes.

β-Catenin is a key regulator of the cadherin-mediated cell-cell adhesion system and an important element in the Wnt signal transduction pathway [59]. Accumulated β-catenin interacts with T-cell factor (Tcf) or lymphoid-enhancer factor (Lef) and translocates to the nucleus, in which it transactivates target genes including c-*myc *and *cyclin D1 *that are the potentially oncogenic [47,60] in the cytoplasm or nucleus as a consequence of mutant *Apc *or β-*catenin *genesis frequently observed in early stages of colorectal carcinogenesis [61,62]. Recently, Girnun *et al*. [21] indicated that a ligand of PPARγ suppresses β-catenin levels and colon carcinogenesis in *Ppar*γ^+/- ^mice treated with AOM. Furthermore, COX-2 is regulated by nuclear β-catenin accumulation [63]. In the current study, treatment with nimesulide, troglitazone, and bezafibrate significantly suppressed β-catenin expression in colorectal adenocarcinomas. Thus, it seems likely that the preventive efficacies of the COX-2 inhibitor (nimesulide) or the PPAR ligands (troglitazone and bezafibrate) against AOM/DSS induced mouse colon carcinogenesis might be mediated, at least in part, by β-catenin down-regulation.

Chemoprevention of cancer might be defined as the deliberate introduction of these selected non-toxic substances into the diet for the purpose of reducing cancer development. In the present study, the mean liver weights of mice in dietary feeding of troglitazone and bezafibrate were significantly increased. However, there were no pathological alterations suggesting toxicity of the drugs examined. It is known that administration of PPARs ligands to rodent exhibit hepatomegaly due to both cellular hypertrophy and hyperplasia [64]. Increase in liver weight by exposure of troglitazone and bezafibrate in this study may be caused by these pathological changes. The estimated daily intakes of nimesulide, troglitazone, and bezafibrate in mice given the diets containing 400 ppm and 500 ppm were approximately 160 mg/kg and 200 mg/kg in the present study. In a direct extrapolation to a 60 kg person, these doses are slightly lower than those of clinical trial [65-67]. These findings may suggest that the efficacy of these agents at dietary dose-levels has a direct practical and translational relevance to human.

## Conclusion

In conclusion, dietary administration of nimesulide, troglitazone, and bezafibrate could effectively suppress colon carcinogenesis induced by AOM and DSS in female ICR mice. Our on-going study on molecular profiles in colonic samples from the current experiment will provide precise molecular mechanisms involved in their inhibitory action in AOM/DSS-induced mouse colon carcinogenesis.

## Abbreviations

AOM, azoxymethane; ACF, aberrant crypt foci; CRC, Colorectal cancer; COX-2, cyclooxygenase-2; DSS, dextran sodium sulfate; FAP, familial adenomatous polyposis; H&E, hematoxylin and eosin; IBD, inflammatory bowel disease; iNOS, inducible nitric oxide synthase; NSAID, non-steroidal anti-inflammatory drug; PPAR, peroxisome proliferator-activated receptor; PCNA, proliferating cell nuclear antigen; ssDNA, single stranded DNA; UC, ulcerative colitis.

## Competing interests

The author(s) declare that they have no competing interests.

## Authors' contributions

HK participated in study design, performed the animal studies, and drafted the manuscript. RS carried out tissue collection and data analysis. SS participated in the histopathological and immunohistological analysis. TT participated in study design, coordination, and manuscript preparation. All authors read and approved the final manuscript.

## Pre-publication history

The pre-publication history for this paper can be accessed here:



## Figures and Tables

**Table 1:** Body, liver, relative liver weights, and length of colon.

Group no.	Treatment	No. of mice	Body weight (g)	Liver weight (g)	Relative liver weight (g/100 g body weight)	Length of colon (cm)
1	AOM/DSS	10	36.6 ± 5.6 ^a^	2.0 ± 0.5	5.5 ± 0.7	11.5 ± 2.1
2	AOM/DSS/0.04% Nimesulide	10	44.8 ± 5.6 ^b^	2.3 ± 0.3	5.3 ± 0.7	12.1 ± 1.3
3	AOM/DSS/0.05% Troglitazone	10	43.6 ± 6.3 ^c^	2.5 ± 0.5 ^d^	5.7 ± 1.0	11.4 ± 1.1
4	AOM/DSS/0.05% Bezafibrate	10	42.4 ± 6.4 ^d^	2.8 ± 0.7^e^	6.6 ± 1.5	12.1 ± 1.4
5	0. 04% Nimesulide	5	39.9 ± 7.3	2.0 ± 0.3	5.0 ± 0.9	12.5 ± 0.7
6	0. 05% Troglitazone	5	39.8 ± 4.0	2.1 ± 0.3	5.2 ± 0.7	11.9 ± 0.9
7	0. 05% Bezafibrate	5	46.8 ± 9.6	2.7 ± 0.5	5.8 ± 0.9	12.6 ± 1.4
8	AOM	5	42.8 ± 2.6	1.9 ± 0.1	4.5 ± 0.4	12.3 ± 1.2
9	DSS	6	34.4 ± 2.8^f^	1.8 ± 0.2	5.2 ± 0.7	12.3 ± 0.6
10	None	5	43.2 ± 6.5	2.0 ± 0.2	4.8 ± 0.8	12.0 ± 0.6

^a ^Mean ± SD.

^b-e ^Significantly different from group 1 by Student's *t*-test (^b ^*P *< 0.005, ^c ^*P *< 0.02, ^d ^*P *< 0.05, and ^e ^*P *< 0.01).

^f ^Significantly different from group 1 by Welch's *t*-test (^f ^*P *< 0.05).

**Table 2:** Incidence and multiplicity of colonic neoplasia.

Group no.	Treatment	No. of mice	Incidence (no. of mice with neoplasms)			Multiplicity (no. of tumors/mice, means ± SD)		
			
			Total	Adenoma	Adeno-carcinoma	Total	Adenoma	Adeno-carcinoma
1	AOM/DSS	10	10/10 (100%)	10/10 (100%)	10/10 (100%)	5.2 ± 3.0	2.1 ± 1.8	3.0 ± 1.8
2	AOM/DSS/0.04% Nimesulide	10	8/10 (80%)	6/10 (60%)^a^	4/10 (40%)^b^	1.8 ± 1.7^b^	1.2 ± 1.3	0.6 ± 1.0^c^
3	AOM/DSS/0.05% Troglitazone	10	9/10 (90%)	9/10 (90%)	4/10 (40%)^b^	2.5 ± 1.8^a^	1.6 ± 1.1	1.2 ± 2.5^a^
4	AOM/DSS/0.05% Bezafibrate	10	8/10 (80%)	7/10 (70%)	6/10 (60%)^a^	2.6 ± 2.5^a^	1.1 ± 1.0^a^	1.8 ± 2.6
5	0.04% Nimesulide	5	0/5 (0%)	0/5 (0%)	0/5 (0%)	0	0	0
6	0.05% Troglitazone	5	0/5 (0%)	0/5 (0%)	0/5 (0%)	0	0	0
7	0.05% Bezafibrate	5	0/5 (0%)	0/5 (0%)	0/5 (0%)	0	0	0
8	AOM	5	0/5 (0%)	0/5 (0%)	0/5 (0%)	0	0	0
9	DSS	6	0/6 (0%)	0/6 (0%)	0/6 (0%)	0	0	0
10	None	5	0/5 (0%)	0/5 (0%)	0/5 (0%)	0	0	0

^a,b,c ^Significantly different from group 1 by Fisher's exact probability test or Student's *t*-test (^a^*P *< 0.05, ^b^*P *< 0.01, and ^c ^*P *< 0.005).

**Table 3:** Incidence of multiplicity colonic mucosal ulcer and dysplasia.

Group no.	Treatment	Incidence (%)				Multiplicity (no. of lesions / mouse, means ± SD)			
		
		Mucosal ulcer	Total dysplasia	Dysplasia with:		Mucosal ulcer	Total dysplasia	Dysplasia with:	
							
				Mild atypia	Severe atypia			Mild atypia	Severe atypia
1	AOM/DSS	40%	90%	80%	50%	0.5 ± 0.7	3.2 ± 1.5	1.4 ± 1.0	1.1 ± 1.3
2	AOM/DSS/0.04% Nimesulide	10%	90%	80%	50%	0.1 ± 0.3	2.2 ± 2.3	1.2 ± 0.9	0.6 ± 0.7
3	AOM/DSS/0.05% Troglitazone	20%	90%	50%	30%	0.3 ± 0.7	2.1 ± 2.2	0.7 ± 0.8	0.8 ± 1.6
4	AOM/DSS/0.05% Bezafibrate	30%	80%	60%	20%	0.4 ± 0.7	1.9 ± 1.8	0.9 ± 1.0	0.4 ± 0.8

**Table 4:** PCNA and apoptosis indices and scores of β-catenin, COX-2, iNOS and nitrotyrosine expression in colonic adenocarcinomas.

Group	Treatment	PCNA-labeling	Apoptotic	Scores for:			
				
no.		index (%)	index (%)	β-Catenin	COX-2	iNOS	Nitrotyrosine
1	AOM/DSS	62.4 ± 13.7^a ^(30)	4.1 ± 1.9 (30)	3.7 ± 0.7 (30)	3.3 ± 0.7 (30)	3.0 ± 1.0 (30)	3.1 ± 0.8 (30)
2	AOM/DSS/0.04% Nimesulide	38.3 ± 11.1 ^b ^(6)	11.8 ± 2.9 ^b ^(6)	2.3 ± 1.0 ^c ^(6)	1.3 ± 0.5 ^b ^(6)	1.3 ± 0.5 ^b ^(6)	1.7 ± 0.5 ^b ^(6)
3	AOM/DSS/0.05% Troglitazone	43.6 ± 9.0 ^b ^(9)	10.0 ± 2.4 ^b ^(9)	2.7 ± 0.7 ^c ^(9)	1.8 ± 0.8 ^b ^(9)	1.6 ± 0.5 ^b ^(9)	1.8 ± 0.6 ^b ^(9)
4	AOM/DSS/0.05% Bezafibrate	40.5 ± 12.7 ^b ^(15)	9.7 ± 2.7 ^b ^(15)	3.0 ± 0.8 (15)	1.8 ± 0.8 ^b ^(15)	1.6 ± 0.6 ^b ^(15)	2.2 ± 1.1 ^b ^(15)

Numbers in parentheses are numbers of lesions examined.

^a ^: means ± SD.

^b,c ^Significantly different from group 1 by Student's *t*-test (^b^*P *< 0.001 and ^c^*P *< 0.05).
